# Ischemia-reperfusion and acidosis induce retrotransposon derepression in the mouse brain

**DOI:** 10.64898/2026.07.31.741803

**Published:** 2026-08-07

**Authors:** Youfang Zhou, Angela Barattini, Xiang-ming Zha, Wenyan Sun

## Abstract

Retrotransposons are repetitive DNA elements normally suppressed through epigenetic mechanisms. In aging and neurodegenerative diseases, abnormal retrotransposon activation occurs and leads to neurotoxicity. However, whether ischemic stroke induces retrotransposon activation remains unclear. Here, we investigated whether cerebral ischemia triggers dysregulation of retrotransposon in the brain. Since brain ischemia leads to tissue acidosis, we further examined whether acidosis contributes to this response. We performed 45 min of transient middle cerebral artery occlusion (tMCAO) followed by reperfusion on adult wild-type mice, then measured expression of retrotransposons LINE-1, IAP, ETn2 and SINE B2 in ipsilateral brain tissue using RT-qPCR. Retroviral GAG protein expression was examined by Western blotting. In vitro, we exposed neuro-2a (N2A) cells to acidic extracellular pH (6.4 or 6.0) and retrotransposon transcripts were analyzed. We found that ischemia-reperfusion increased expression of IAPEz-gag and ETn2 in ipsilateral ischemic brain tissue at 24 h, with stronger induction of LINE-1 Orf1, IAPEz-gag and B2 in infarct than peri-infarct brain regions. Western blotting analysis revealed dynamic changes in GAG proteins, with no detectable changes at 6 h of reperfusion followed by reduced levels of the precursor Pr65 and mature capsid p30 products at 24 h. In N2A cells, extracellular acidosis induced time-dependent increases in LINE-1 Orf1, IAPEz-gag and B2 transcripts. These findings identify retrotransposon dysregulation as a molecular feature of ischemic brain injury and provide a framework for further investigating its role in stroke pathology.

## Full Text Availability

The license terms selected by the author(s) for this preprint version do not permit archiving in PMC. The full text is available from the preprint server.

